# Biomarkers of postharvest resilience: unveiling the role of abscisic acid in table grapes during cold storage

**DOI:** 10.3389/fpls.2023.1266807

**Published:** 2023-09-29

**Authors:** Ángela Navarro-Calderón, Natalia Falagán, Leon A. Terry, M. Carmen Alamar

**Affiliations:** Plant Science Laboratory, Cranfield University, Cranfield, United Kingdom

**Keywords:** senescence, ABA, ethylene, 1-methylcyclopropane, table grapes, postharvest

## Abstract

Table grapes are considered non-climacteric fruit, not showing a rapid increase in respiration rate and ethylene production during ripening. Previous research has suggested that abscisic acid (ABA) may have a more crucial role in grape postharvest behaviour. This study aimed to identify biomarkers of postharvest resilience and flavour life of imported table grapes. An experiment was designed to determine i) the role of ABA and catabolites on grape berry senescence; ii) the spatial distribution of these hormones within the grape berry, and iii) the effect of 1-MCP and storage temperature on its postharvest quality. Hence, the use of an ethylene inhibitor, 1-methylcyclopropane (1-MCP), during table grape storage was investigated. Table grapes (*Vitis vinifera* L.) cv. ‘Krissy’ were subjected to i) control (untreated); and ii) 1-MCP (1 µL L^-1^; 12 hours; 15°C) and stored under two scenarios: i) 15 days at 0.5°C, followed by five days at 5.5°C to simulate shelf-life; and ii) 20 days at 5.5°C to simulate a higher storage temperature followed by shelf-life. Physiological (i.e. mould incidence, skin colour, firmness, respiration rate) and biochemical analysis (i.e. individual sugars, organic acids, abscisic acid and catabolites) were performed. Grapes subjected to 5.5°C showed significantly higher mould incidence at the end of the shelf-life compared to 0.5°C storage temperature (12.6% *vs*. 3.1%). Also, and for the first time, the spatial distribution of ABA during the senescence of table grapes was profiled; the distal section had three times more ABA and metabolites than the proximal. We demonstrated that senescence processes were initiated after a significant increase in respiration rate (from 1 to 2.8 mL CO_2_ kg^-1^ h^-1^), and that ABA could be considered a biomarker for table grapes senescence, since an ABA peak preceded the increase in respiration rate, mould incidence, organic acids, and sucrose hydrolysis during postharvest storage; and coincided with a decrease in berry firmness. These findings are of significant importance for the industry as understanding how ABA regulates both senescence processes and quality changes during postharvest cold storage of tables grapes can improve the consistency and reduce waste and consumer complaints.

## 1 Introduction

Table grapes follow a non-climacteric ripening pattern, and thus do not show a burst in respiration and ethylene production at the onset of ripening (Zenoni et al., 2023). However, it has been reported that ethylene is somehow involved in regulating grape ripening processes, such as the decrease in acids and the accumulation of sugars and anthocyanins during berry development (Yan et al., 2023). Nonetheless, many studies have highlighted a more influential role of ABA on these ripening processes (Sun et al., 2010; McAtee et al., 2013; Pilati et al., 2017; Coelho et al., 2019; Gao-Takai et al., 2019; Zenoni et al., 2023).

There are three ABA peaks, which occur during grape berry growth, at ripening, and at postharvest senescence, the latter being an irreversible process if a certain ABA threshold is achieved. ABA can be esterified to ABA-GE and stored in the vacuoles to respond to stresses, hydroxylated to 7’ -, 8’ -, or 9’-OH-ABA, or isomerised to PA or DPA (Jia et al., 2017). Although some research has been done on ABA and ABA metabolites during ripening and senescence in fruits like cherries (Serradilla et al., 2019) and strawberries (Tosetti et al., 2020), little is known about the role of ABA and the different ABA catabolites during grape berry postharvest senescence. Previous research has highlighted the role of phytohormones as regulators of defence mechanisms against pathogens responsible for e.g. decay (Amaro et al., 2023). In particular, ABA has been found to be associated to grapevine susceptibility towards pathogens such as *Erysiphe necator* (Coelho et al., 2019).

The ethylene antagonist 1-methylcyclopropene (1-MCP) has been widely used in postharvest studies to better understand the ripening process of fresh produce (Watkins, 2006). However, there is a lack of information on the effect of 1-MCP on grapes postharvest quality and senescence. Moreover, the results obtained from the few published studies are not conclusive. Wang et al. (2019) applied 1-MCP immediately after harvest and they found that 1-MCP was able to reduce decay, minimised abscission and inhibited raquis browning in table grape branches (‘Kyoho’ and ‘Yongyou NO.1’). In another study, Bellincontro et al. (2006) investigated the effect of 1-MCP on stored grapes. Their results showed that 1-MCP lowered ethylene production, whilst no change in grape berry respiration rate (RR) was observed. Moreover, 1-MCP-treated berries showed a higher incidence of grey mould and a significant loss of terpenols and esters. More recently, Silva et al. (2013) applied different doses of 1-MCP (0, 500, 1,000 and 2,000 nL L^-1^ for 12 hours) to stored berries and found that the treatment maintained the ascorbic acid and total anthocyanins contents without affecting berry firmness and decreased berry drop as the doses increased.

Therefore, despite the efforts made to understand the mechanisms behind the ripening and senescence of non-climacteric fruit, the role of the different ripening hormones remains unclear. The objective of this study was three-fold: i) to understand the role of ABA and ABA catabolites on grape berry senescence; ii) to study the spatial distribution of these hormones within the grape berry during senescence and its effect on berry quality, and iii) to evaluate the effect of 1-MCP and storage temperature on the postharvest quality of ‘Krissy’ table grapes.

## 2 Materials and methods

### 2.1 Plant material

The experiment was conducted on grapes (*Vitis vinifera* L.) from a commercial vineyard of drip irrigated 3-year-old ‘Krissy’ vines grafted onto Paulsen 1103 (3.5 m × 3 m spacing) located in Murcia (SE Spain).

The grape clusters (n = 200) were monitored during the 2018 growing season and harvested at their optimal commercial maturity according to market standards. The samples were then transported to the Plant Science Laboratory at Cranfield University (UK) by refrigerated lorry (0.5°C and 85% RH) within five days of harvest as per standard commercial practice.

### 2.2 Experimental design

Grape clusters showing decay or mechanical damage were discarded, and sound fruit was placed in cold storage (0.5°C and 85% RH) overnight to acclimatise. Then, two treatment groups were considered: i) control (untreated); and ii) 1-MCP, where one hundred grape clusters were placed inside 100 L hermetically sealed boxes and treated with 1-MCP (1 µL L^-1^) for 12 hours at 15°C, as described in Foukaraki et al. (2016).

Following the 1-MCP application, two storage scenarios were considered: i) scenario 1: 15 days at 0.5°C and 85% RH as per normal storage conditions, followed by five days at 5.5°C and 85% RH to simulate shelf-life; and ii) scenario 2: 20 days at 5.5°C and 85% RH to simulate a higher storage temperature followed by shelf-life.

Three replicates per treatment group and storage scenario were analysed every five days. Each replicate consisted of three grape clusters from which thirty random berries were analysed (n = 90).

### 2.3 Physiological analysis

#### 2.3.1 Mould incidence, berry skin colour and berry firmness

Mould incidence was recorded as the percentage of mouldy berries per replicate, according to Youssef et al. (2019). Afterwards, grape skin colour was determined using a hand-held tristimulus colourimeter with a D65 illuminant and 8 mm light aperture (model CR-400 Chroma Meter, Konica Minolta Inc., Cheshire, UK). The colourimeter was calibrated with a white plate, and it provided the lightness (L*), chroma index, and Hue angle values of the samples. These parameters are widely used as a measure of ripeness (Conesa et al., 2016).

The firmness of individual berries was determined using a uniaxial testing machine calibrated with a 5 N cell (Instron, model 5542, MA, USA). Maximum compressive load in Newtons (N) was measured by compressing the berry 1 mm deep with a 4 mm diameter probe at a speed of 100 mm min^-1^ (Alamar et al., 2017).

#### 2.3.2 Respiration rate

Respiration Rate (RR) was measured as described in Collings et al. (2018). Samples were placed in 3 L airtight glass jars and dry clean air (300 mL min^-1^) was passed through the jars into a Sable Respirometry System (model 1.3.8 Pro, Sable Systems International, Las Vegas, USA). Each sample jar (n = 3 replicates per treatment) was measured for 2 min to achieve a stable reading. An empty jar (continuous air for 1 min) was used as a baseline to avoid cross-contamination between treatment measurements. The rate of CO_2_ production was expressed in mL kg^-1^ h^-1^.

### 2.4 Biochemical analysis

For biochemical analysis, berries were cut transversely at *ca.* one third from the top of the berry. Before biochemical analysis, the samples were snap-frozen in liquid nitrogen and placed in a -50°C CoolSafe freeze-dryer (Scanvac, Labogene, Denmark) for seven to ten days. After this, the freeze-dried samples were ground and kept at -40°C until analysis (García-Pastor et al., 2021b).

#### 2.4.1 Individual sugars

Extraction and analysis of individual sugars (glucose, fructose, and sucrose) were done following Davis et al. (2007). Briefly, 150 mg of freeze-dried fruit samples were extracted with 3 mL of 62.5% (v/v) aqueous High Performance Liquid Chromatography (HPLC)-grade methanol in a water bath at 55°C for 15 min, vortexing every 5 min for 30 s. The extract was then filtered through 0.2 μm PTFE filters and stored in 2 mL HPLC vials at -40°C until analysis. Before analysis, the extracts were diluted 1:9 with HPLC grade water.

Detection was performed using a Refractive Index Detector (RID) coupled to an Agilent 1200 series HPLC system (Agilent Technologies, Germany) with a Phenomenex Rezex RCM monosaccharide Ca^+2^ (8%) ion exclusion column (300 mm x 7.8 mm), 8 μm particle size, fitted with a Phenomenex Carbo Ca^+4^ mm x 3 mm guard column (Phenomenex, CA). The column oven temperature was set at 80°C and the refractive index detector at 50°C. The mobile phase used was HPLC-grade water at a flow rate of 0.6 mL min^-1^, and the autosampler injection volume was 20 μL. A 6-point calibration curve ranging from 0.05 to 2.5 mg mL^-1^ was used for quantification, and the results were expressed as mg g^-1^ dry weight (DW).

#### 2.4.2 Organic acids

Analysis of non-volatile organic acids was done as described in Terry et al. (2007) with slight modifications. Briefly, freeze-dried samples were dissolved in 3% aqueous metaphosphoric acid, kept at room temperature for 10 min, filtered through 0.2 μm cellulose filters into HPLC vials, and analysed immediately after extraction.

The acids content was determined using an Agilent 1200 series HPLC system with an Alltech Prevail Organic Acid 250 mm x 4.6 mm, 5 μm particle size column fitted with an Alltech Prevail Organic Acid 7.5 mm x 4.6 mm, 5 μm particle size guard column (Alltech, CA, USA). The column oven temperature was set at 35°C, and the compounds were detected by a diode array detector (DAD) set at 210 nm. The mobile phase used was 25 mM KH_2_PO_4_ HPLC-grade water at a flow rate of 1.5 mL min^-1^, and the autosampler injection volume was 20 μL. A 5-point calibration curve ranging from 0.05 to 1 mg mL^-1^ with a mixture of tartaric and malic acids was used for quantification, and the results were expressed as mg g^-1^ DW.

#### 2.4.3 Abscisic acid and catabolites

ABA and ABA catabolites concentration was determined by following Serradilla et al. (2019) with modifications. Freeze-dried samples (5 mg) were mixed with 500 μL of methanol/formic acid/water (60:5:35, v/v) and 10 μL of the internal standards mix (100 ng mL^-1^) in a 1.5 mL Eppendorf tube, and shaken in a Star Beater (R&L Slaughter Ltd., Essex, UK) at 30 Hz for 2 min. The internal standard mix was made of the labelled forms of the compounds: (-)-5,8'8'8'-d4-abscisic acid (d4-ABA); (-)-7',7',7'-d3-phaseic acid (d3-PA); (-)-7',7',7'-d3-dihydrophaseic acid (d3-DPA); (+)-4,5,8',8',8'd5-abscisic acid glucose ester (d5-ABA-GE); (±)-5,8',8',8'-d4-7'-dihydroxy-ABA (d4−7 -OH-ABA). The tubes were then placed in dry ice and kept in darkness for 20 min. Afterwards, the tubes were centrifuged at 4°C and 14,000 rpm for 10 min, then transferred to 15 mL falcon tubes and freeze-dried overnight. The extracts were reconstituted with 500 μL of acetonitrile/formic acid/water (10:0.1:89.9, v/v), vortexed for 1 min, sonicated for 1 min, and centrifuged at 4°C and 4,500 rpm for 1 min. The samples (20 μL) were then injected on a Phenomenex Luna C18 100 mm x 2 mm, 3 μm with guard column at 40 °C, and analysed by an Agilent 1200 series HPLC system coupled to a Q-Trap 6500 mass spectrometer (AB Sciex, MA, USA). The concentration of ABA and ABA metabolites was quantified based on Morris et al. (2018) methodology. A 6-point calibration curve ranging from 0.1 to 100 ng mL^-1^ was used for quantification, and the results were expressed as ng g^-1^ DW.

### 2.5 Statistical analysis

All statistical analyses were carried out using Statistica for Windows version 13 (Dell Inc., USA). Data were subjected to normality tests and outliers were removed if necessary. An analysis of variance (ANOVA) was performed to test the experimental hypotheses. When significant differences were found, the data were subjected to a Fisher’s *post-hoc* test. Least significant differences (LSD) were calculated for each analysis (*p* < 0.05) and shown on the graphs when the interaction was significant. When factors were significant, LSD values were included in the corresponding caption for comparison. The differences between scenario/temperature, treatment (± 1-MCP), storage time and berry section (for biochemical analysis) were studied. Figures were plotted in SigmaPlot 14 (Systat Software Inc., USA).

## 3 Results

### 3.1 Mould incidence, berry firmness, and berry skin colour

**Figure 1:**
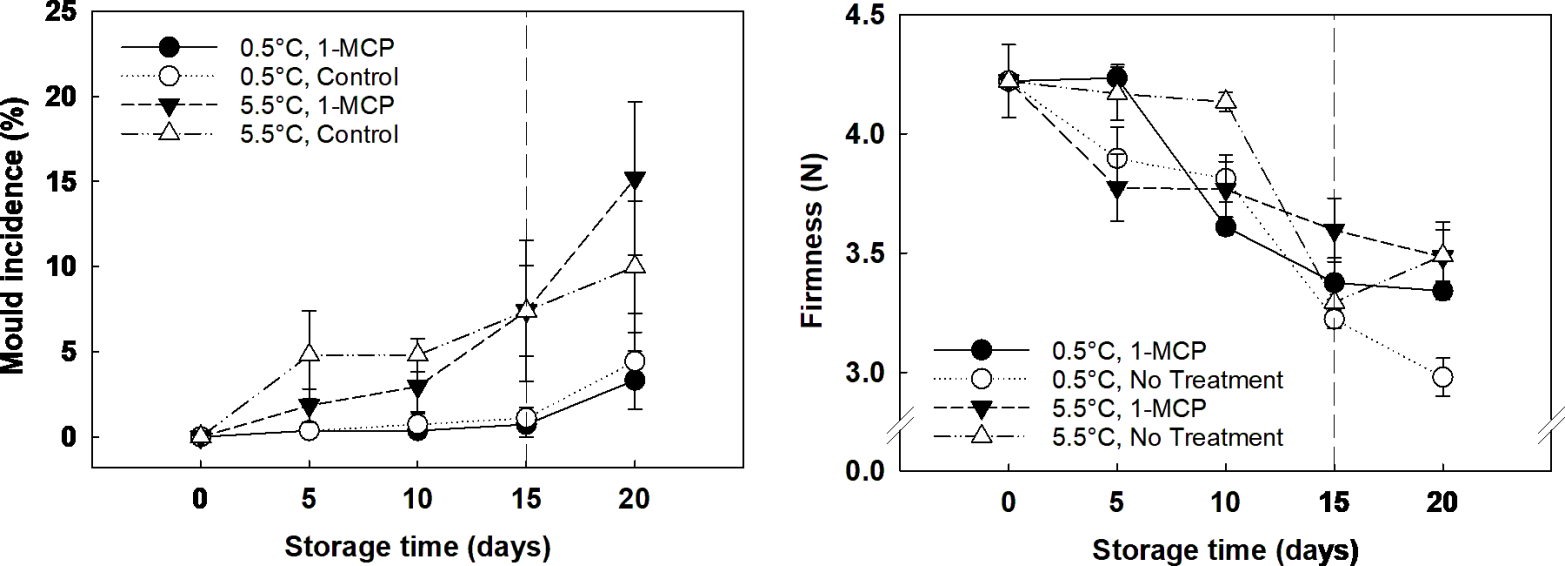
Mould incidence (%; left) and firmness (N; right) of ´Krissy´ grapes. Table grapes were treated on arrival (5 days postharvest) with 1-MCP (1 µL L^-1^ for 12 h at 15°C [1-MCP]) or without 1-MCP (air [control]) prior to storage and subjected to two postharvest storage scenarios: i) 15 days at 0.5°C and 85% RH, followed by 5 days at 5.5°C, and ii) 20 days at 5.5°C and 85% RH. The vertical stripped line shows when all samples were stored at 5.5°C. Data represents means (n = 90 berries) ± standard error. LSD_0.05 [mould incidence]_ temperature x storage time = 3.71. LSD_0.05 [firmness]_ storage time = 0.31.

Mould incidence in grapes was solely affected by storage temperature, being five times more severe in grapes stored at 5.5°C than the incidence found on grapes stored at 0.5°C, which did not show any decay until the end of storage. In the case of firmness, it was observed that storage time was the key factor involved, showing a decline during storage even for berries subjected to 1-MCP treatment (Figure Captions **Figure 1**). Chroma index progressively decreased over storage time (**Supplementary Figure 1**), and Hue angle significantly increased at the end of storage, with berries acquiring a dull, dark red skin colour. These changes were linked to storage time, playing 1-MCP and temperature a non-significant role.

### 3.2 Respiration rate

**Figure 2:**
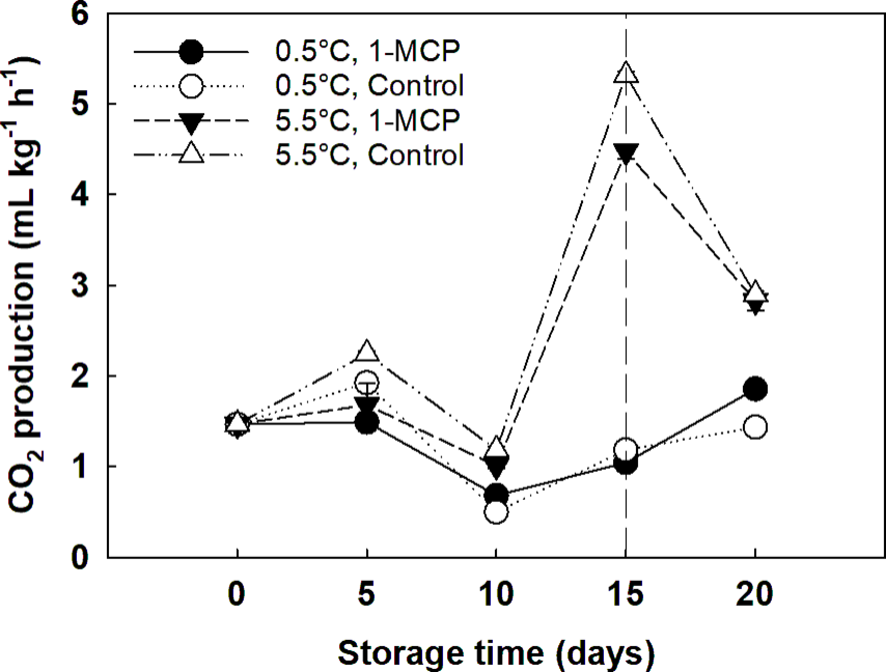
Respiration rate (RR) expressed as CO2 production (mL kg^-1^ h^-1^) of ´Krissy´ grapes. Table grapes were treated with 1-MCP (1 µL L^-1^ for 12 h at 15°C [1-MCP]) or without 1-MCP (air [control]) prior to storage and subjected to two postharvest storage scenarios: i) 15 days at 0.5°C and 85% RH, followed by 5 days at 5.5°C, and ii) 20 days at 5.5°C and 85% RH. The vertical stripped line shows when all samples were stored at 5.5°C. Data represents means (n = 90 berries) ± standard error. LSD_0.05_ for the interaction temperature x storage time = 0.3; LSD_0.05_ for the interaction treatment x storage time = 0.3.

The treatment with 1-MCP significantly decreased the RR of the grape berries stored at 5.5°C compared to control (2.3 and 2.6 and mL CO_2_ kg^-1^ h^-1^, respectively). However, 1-MCP did not affect grapes stored at 0.5°C. The CO_2_ production of grape berries significantly decreased (p = 0.000000) at the beginning of the storage, from 1.5 to 0.8 mL CO_2_ kg^-1^ h^-1^ at day 0 and day 10, respectively. Then, RR significantly increased to 3.0 mL CO_2_ kg^-1^ h^-1^ at day 15. Storage temperature was the main effect for RR: grapes stored at 5.5°C showed significantly higher RR (p = 0.000000; 2.5 mL CO_2_ kg^-1^ h^-1^) than grapes stored at 0.5°C (1.4 mL CO_2_ kg^-1^ h^-1^; **Figure 2**).

### 3.3 Individual sugars

**Figure 3:**
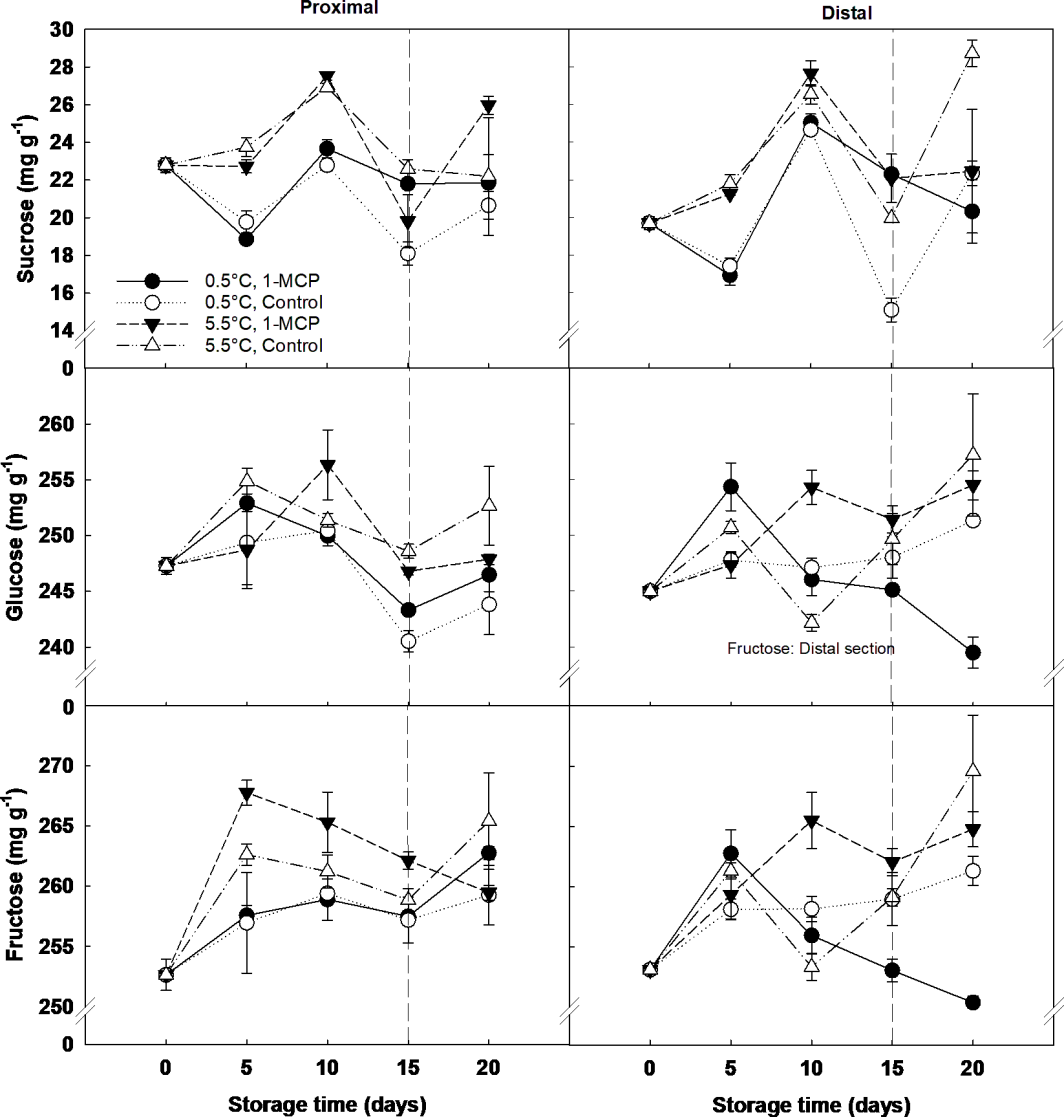
Individual sugars (sucrose, fructose and glucose) expressed as mg g^-1^ DW in the proximal (left) and distal (right) sections of ´Krissy´ grapes. Table grapes were treated with 1-MCP (1 µL L^-1^ for 12 h at 15°C [1-MCP]) or without 1-MCP (air [control]) prior to storage and subjected to two postharvest storage scenarios: i) 15 days at 0.5°C and 85% RH, followed by 5 days at 5.5°C and ii) 20 days at 5.5°C and 85% RH. The vertical stripped line shows when all samples were stored at 5.5°C. Data represents means (n = 90 berries) ± standard error. LSD_0.05 [sucrose, glucose, fructose]_ for storage time =1.51, 3.26, 3.28, respectively. LSD_0.05 [sucrose, glucose, fructose]_ for temperature =10.97, 1.98, 1.99, respectively.

The main sugars found in the grape berry were fructose and glucose, while the concentration of sucrose was ten times lower (**Figure 3**). Storage time and temperature were the key factors affecting sugar content, whilst 1-MCP only had a significant effect at the end of shelf-life. At this time, the distal section of the treated berries stored at 0.5°C showed a significantly lower content of fructose and glucose than the rest of the samples. Sucrose concentration significantly increased from day 5 (20 mg g^-1^ DW) to day 10 (26 mg g^-1^ DW) of storage, and then decreased to similar values of those recorded at day 5 for both storage temperatures and sections.

### 3.4 Organic acids

**Figure 4:**
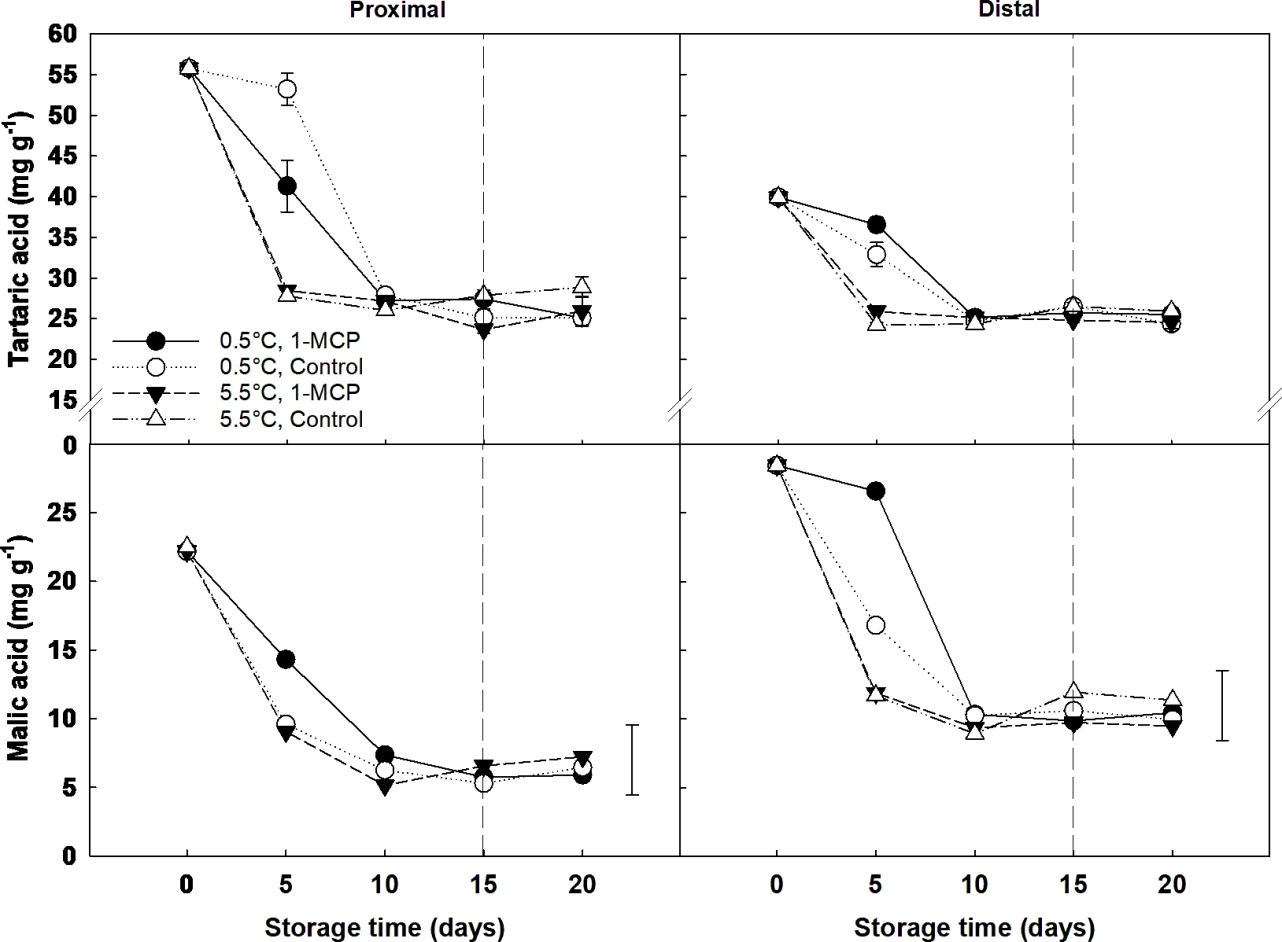
Organic acids (tartaric and malic acids) expressed as mg g^-1^ DW in the proximal (left) and distal (right) sections of ´Krissy´ grapes. Table grapes were treated with 1-MCP (1 µL L^-1^ for 12 h at 15°C [1-MCP]) or without 1-MCP (air [control]) prior to storage and subjected to two postharvest storage scenarios: i) 15 days at 0.5°C and 85% RH, followed by 5 days at 5.5°C and ii) 20 days at 5.5°C and 85% RH. The vertical stripped line shows when all samples were stored at 5.5°C. Data represents means (n = 90 berries) ± standard error. LSD_0.05 [tartaric acid]_ for the interaction temperature x section x storage time = 4.35; LSD_0.05 [malic acid]_ for the interaction temperature x treatment x section x storage time = 5.09.

The concentration of both tartaric and malic acid experienced a significant decrease from day 0 to day 10, independent of the berry section sampled, and then remained constant until the end of shelf-life. Significant differences between temperatures and treatments were only observed at day 5, with treated grapes stored at 0.5°C showing a higher level of malic acid than the rest of the samples. The effect of 1-MCP on the tartaric and malic acid content was not significant at 5.5°C; however, it did affect the organic acids content at the beginning of storage when the berries were kept at 0.5°C. Thus, malic acid levels on day 5 were significantly higher for treated grapes stored at 0.5°C than control, and this difference was more pronounced in the distal section of the berries. In contrast, the application of 1-MCP resulted in a significant decrease in the overall content of tartaric acid in table grape berries during the first five days of storage at 0.5°C. Yet this differences between treatments disappeared from day 10 onwards. When looking at the spatial distribution of organic acids, a higher tartaric acid concentration was found in the proximal section at the beginning of storage - day 5 - (average *ca*. 40 mg g^-1^ DW) compared to the distal section (average *ca*. 28 mg g^-1^ DW); while malic acid showed the opposite trend - more concentrated in the distal section (*ca*. 18 *vs*. 12 mg g^-1^ DW, respectively) (**Figure 4**).

### 3.5 Abscisic acid and catabolites

**Figure 5:**
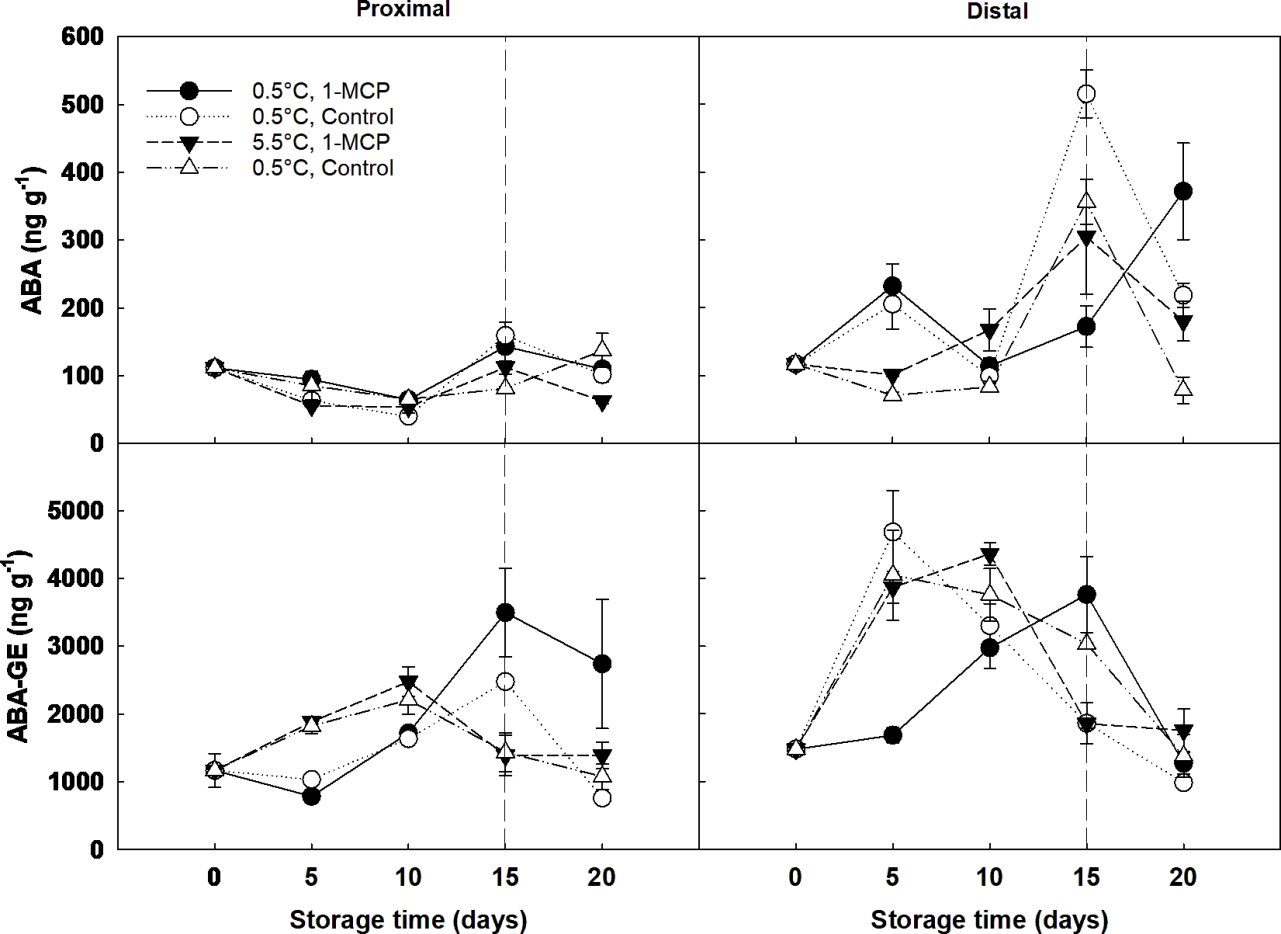
Abscisic acid (ABA) and the ABA metabolite ABA-glucose ester (ABA-GE) expressed as ng g^-1^ DW in the proximal (left) and distal (right) sections of ´Krissy´ grapes. Table grapes were treated with 1-MCP (1 µL L^-1^ for 12 h at 15°C [1-MCP]) or without 1-MCP (air [control]) prior to storage and subjected to two postharvest storage scenarios: i) 15 days at 0.5°C and 85% RH, followed by 5 days at 5.5°C, and ii) 20 days at 5.5°C and 85% RH. The vertical stripped line shows when all samples were stored at 5.5°C. Data represents means (n = 90 berries) ± standard error. LSD_0.05 [ABA]_ for the interaction storage time x treatment x section = 102.22. LSD_0.05 [ABA-GE]_ for the interaction storage time x section = 956.36; for the interaction storage time x temperature = 102.22.

Overall, ABA and its catabolites significantly increased throughout cold storage and decreased by the end of shelf-life (**Figure 5**; **Supplementary Figure 2**). Grapes stored at 5.5°C had 20% lower ABA and 15% higher ABA-GE content than grapes stored at 0.5°C. The distal section of the berries showed a significantly higher (3-fold) ABA content than the proximal section. However, the ABA content in the distal section of 1-MCP-treated grapes was approximately half (238 ng g^-1^ DW) of that found in control berries (435 ng g^-1^ DW) at the end of storage (**Figure 5**). Phaseic acid was not detected in our samples.

## 4 Discussion

### 4.1 1-MCP application increases mould incidence in ‘Krissy’ table grapes

The ethylene antagonist 1-MCP is a compound that inhibits ethylene perception by binding to and hence inactivating the ethylene binding protein (EBP; Kyaw et al., 2023). Previous studies have shown how 1-MCP can inhibit senescence related processes in non-climacteric fruits such as grapes (Li et al., 2016a) and sweet cherries (Serradilla et al., 2019). Bellincontro et al. (2006) demonstrated that a single dose of 1-MCP (1 mg L^-1^) applied for 15 h at 20°C in 300 L boxes during postharvest storage reduced ethylene production yet only for two days after treatment application. In addition, there was an increase in grey mould incidence after six days of storage at 20°C (Bellincontro et al., 2006).

It has been previously reported that ethylene is required to upregulate defence-related genes against fungal attack in Arabidopsis (Ferrari et al., 2003). Therefore, blocking ethylene production with 1-MCP could result in a higher mould incidence at the end of shelf life, as the conditions for mould growth are more favourable because of berry firmness loss and degradation of organics acids and sucrose. Moreover, the higher mould incidence found at the end of the storage in 1-MCP treated grapes was mirrored by lower concentrations of fructose and glucose in the distal section of the berry. It can be hypothesised that these monosaccharides were being used as substrate by the fungi. Further research should be carried out to validate this hypothesis. Other ethylene removal methods for the storage and transportation of fruit and vegetables have been reviewed recently (Ying et al., 2021). Thus, while 1-MCP inhibits ethylene, other technologies like ozone, low-temperature catalytic oxidation, and plasma catalysis act by eliminating ethylene. For instance, Feliziani et al. (2014) demonstrated that storing ‘Crimson Seedless’ table grapes in chambers containing 0.1 μL L^-1^ ozone or higher at 2°C for three weeks reduced grey mould incidence by 65% after 5 to 8 weeks of storage.

In the study herein, treated grapes stored at 5.5°C were highly affected by mould incidence at the end of shelf-life. This result agrees with the above-mentioned study (Bellincontro et al., 2006) and with previously published research on non-climacteric fruit like strawberry (Bower et al., 2003). It has been demonstrated previously that local resistance to *Botrytis cinerea* (grey mould) in Arabidopsis requires the upregulation of defence genes that use ethylene as a secondary messenger (Ferrari et al., 2003). Therefore, blocking ethylene production with 1-MCP could be stopping the expression of these defence genes and, as a result, decreasing the crop resistance to a fungal attack.

### 4.2 1-MCP treatment delayed sucrose hydrolysis and the decline in the organic acids during table grape cold storage

Regarding RR, table grapes stored at 5.5°C experienced a significant increase in CO_2_ production from day 10 to day 15, which was lower in 1-MCP treated grapes than in control. It has been previously proposed that the increase in RR at the onset of ripening occurs due to a change in the berries’ metabolism and that sugars, and not malate, are the primary substrate used for respiration (Famiani et al., 2014). This hypothesis is confirmed by the results seen here, which showed that a lower RR due to the 1-MCP treatment delayed both the hydrolysis of sucrose into fructose and glucose and the decline in the organic acid content compared to untreated grapes. In contrast to these results, Bellincontro et al. (2006) and Li et al. (2016b) found that 1-MCP applied after harvest did not significantly affect the RR of ‘Aleatico’ wine grapes and ‘Thompson Seedless’ white table grapes, respectively. However, it is necessary to highlight that Li et al. (2016b) only measured the RR of five berries clipped off at the end of the pedicel after three and six days from applying the 1-MCP treatment, while in this study the RR of three whole bunches per condition was measured every five days for 20 days. Therefore, while 1-MCP delays the increase in RR and the degradation of organic acids and sugars in table grapes stored at 5.5°C, the treatment is not able to counteract the effect of a higher storage temperature, which results in a higher mould incidence and, hence, the unsuitability of the grapes to be commercialised and accepted by the end consumer. According to our results, the use of 1-MCP has not been proved to have a positive effect on extending the postharvest life of table grapes, therefore, it is recommended to evaluate the effect of other ethylene removal techniques like the ones mentioned above.

### 4.3 ABA metabolites: the hidden players in grape berry ripening and stress responses

#### 4.3.1 Abscisic acid accumulation can regulate grape berry metabolism

ABA is known to regulate plant growth and developmental processes, such as cell division, seed dormancy, maturation, ripening, and abscission, as well as stress responses to drought, cold, or pathogen attack (Finkelstein, 2013; Jia et al., 2017; Coelho et al., 2019). While a few studies have investigated the profile and role of ABA and its catabolites during the preharvest stages of grape berry development and ripening, less attention has been given to the hormonal profile of berries during postharvest and senescence. According to Coombe and Hale (1973), the onset and rate of berry ripening are a function of ABA accumulation. The authors suggested that berry ripening starts once ABA accumulates to a certain level (*viz*. 62 ng g^-1^ fresh weight [FW]), below which the berry will not ripen. In the literature, there is a considerable variation in the value of this peak and the moment in berry development when it appears. For instance, Coombe and Hale (1973) observed that ‘Doradillo’ grape veraison started with an ABA concentration of 62 ng g^-1^ FW, which then increased to 212 ng g^-1^ FW 13 days after veraison. In contrast, Sun et al. (2010) reported an ABA peak of 500 ng g^-1^ FW two days after ‘Muscat Hamburg’ grape veraison and a second ABA peak at similar levels six days after harvest after which the berries started to senesce. In a study by Owen et al. (2009), the ABA concentration in the pulp of immature ‘Merlot’ grape berries was 4,000 ng g^-1^ DW, while mature berries harvested at commercial maturity had an ABA content of 400 ng g^-1^ DW. Moreover, the authors found that ABA was catabolised to form ABA-GE by conjugation instead of producing DPA by oxidation at the onset of veraison. However, the mentioned study did not quantify ABA or its catabolites during the postharvest berry senescence.

In this work, ABA accumulation coincided with a decrease in the organic acid content and firmness of the berries and an increase in berry respiration rate, sugar hydrolysis, and disease incidence, which are factors determining quality acceptance for the final consumer (Wolf, 2002; Jianying et al., 2014). These results suggest that the grape berries started to senesce after reaching an ABA concentration higher than 100 ng g^-1^ DW and that ABA played a role in regulating grape berry metabolism during storage and shelf-life. The crosstalk between ABA and sucrose in regulating fruit ripening processes has been recently reviewed (Durán-Soria et al., 2020; Alferez et al., 2021). Jia et al. (2017) found that treatments with exogenous ABA and sucrose regulate berry ripening of ‘Fujiminori’, a black table grape, and that sucrose can induce the ABA synthesis gene expression. According to Luo et al. (2020), ABA and sucrose play a synergistic role in regulating the ripening of strawberry, a non-climacteric produce. Our results were not conclusive in this area; even though we found a negative correlation between sucrose and ABA, there was not enough evidence to consider this correlation as proof for signalling the triggering of senescence processes.

In addition to regulating sugar levels, ABA is involved in the biosynthesis of secondary metabolites like anthocyanins, which are pigments that give red, purple, and blue colours to plants (Owen et al., 2009; Zhang et al., 2014). For this reason, ABA is applied to coloured grape varieties during berry maturation to improve skin colour development by enhancing the accumulation of anthocyanin biosynthetic enzyme genes (Yamamoto et al., 2015; Domingues Neto et al., 2017). In this study, an increase in the fructose and glucose content coincided with darker berry colour and a higher ABA content by the end of storage and shelf-life, confirming the role of sugars in the stimulation of pigmentation (Durán-Soria et al., 2020). Darker berry colour is related to higher consumer acceptability, and therefore, the understanding of ABA metabolism and its interaction with pigment development is key for the grape industry.

#### 4.3.2 ABA metabolites: unravelling their role in ripening and stress response

ABA-GE concentration was ten times higher than ABA, playing an important role in grape berry ripening, especially on stressed fruit (Zarrouk et al., 2016). The highest peak in ABA-GE accumulation occurred for berries subjected to 1-MCP at 0.5 C with the jump of temperature from storage to shelf life stages, showing an abiotic stress as an adjustment to the changing environment. The general trend for ABA-GE concentration was increasing, linking to berry development (Zarrouk et al., 2016). It has been demonstrated that ABA-GE can regulate ABA accumulation; this is interesting because the increase of ABA concentrations follows a previous increase in ABA-GE concentrations, suggesting that ABA-GE can play a role in ABA synthesis through β-glucosidase in agreement with e.g., López-Carbonell et al. (2009) and Xu et al. (2014).

ABA is stored in the form of ABA-GE in the vacuoles and the apoplast during the early berry developmental stages, being released to the endoplasmic reticulum under water stress or dehydration (Finkelstein, 2013; Jia et al., 2017). Thus, it could be hypothesised that the proximal section would have a higher hormonal content due to dehydration in the abscission zone, as ripening related changes in the cell wall undergo from the formation of a dehiscence zone to the softening of the flesh tissue (McAtee et al., 2013). Contrastingly, here it was found that the concentration of ABA and its metabolites, mainly ABA-GE, was significantly higher (3-fold) in the distal section when compared to the proximal section, confirming the hypothesis of a different hormonal distribution within the grape berry during senescence. Two facts could explain this: firstly, the distal section of the berries had a darker colour than the proximal, and secondly, it was observed that this section also presented a higher mould incidence during the last stages of cold storage and shelf-life.

Conversely, DPA concentration was ten times lower than ABA in agreement with a previous study by García-Pastor et al. (2021a) on ‘Magenta’ table grape. DPA showed an increasing trend, particularly during shelf life as seen in Pogány et al. (2020).

High levels of DPA have been observed during the early developmental stages (Owen et al., 2009). Therefore, a low level of this hormone could potentially indicate an advanced level of ripening or senescence. A larger sample size and a deeper dive in molecular biology pathways could help identify the involvement of DPA in ripening and stress responses (**Supplementary Figure 2**).

## 5 Conclusions

The concomitant increase of ABA, respiration rate, and sucrose observed at the end of cold storage confirmed the crucial role of ABA in regulating processes associated with the senescence of the non-climacteric grape berry. 1-MCP delayed the degradation of sugars and organic acids, key contributors to flavour life and therefore becoming a good tool to maintain organoleptic traits. However, whether respiration or the changes in endogenous ABA concentration are the triggers of the different senescence mechanisms undergone in the grape berry needs further investigation. It is also recommended that these studies focus their attention on the distal section of the berries, as it has shown a higher hormonal content than the proximal part. The benefit of ethylene antagonists like 1-MCP on non-climacteric produces during postharvest storage is not entirely evident yet, and further research is suggested.

## Data availability statement

The datasets generated and analysed in this study can be found in CORD using the following link http://doi.org/10.17862/cranfield.rd.23727189.

## Author contributions

MA: Conceptualization, Data curation, Methodology, Supervision, Visualization, Writing – review & editing. NF: Writing – review & editing. LT: Conceptualization, Funding acquisition, Resources, Supervision, Writing – review & editing. ÁN: Conceptualization, Data curation, Formal Analysis, Methodology, Visualization, Writing – original draft, Writing – review & editing.
