# Supplementary material for: Biomarkers of postharvest resilience: unveiling the role of abscisic acid in table grapes during cold storage
Source: Front Plant Sci. 2023 Sep 29;14:1266807. doi: 10.3389/fpls.2023.1266807 (PMC10570816; doi:10.3389/fpls.2023.1266807)
Supplement: Supplementary file 1 [file DataSheet_1.docx]

Supplementary Material

Biomarkers of postharvest resilience: unveiling the role of abscisic acid in table grapes during cold storage

**Ángela Navarro-Calderón, Natalia Falagán, Leon A. Terry, M. Carmen Alamar***

*** Correspondence:** [m.d.alamargavidia@cranfield.ac.uk](mailto:m.d.alamargavidia@cranfield.ac.uk)

**Supplementary Figure 1.** **CIELab colour parameters (Lightness [L*], Chroma index [C*], and Hue angle [H^o^]) of ´Krissy´ grapes.** Table grapes were treated with 1-methylcyclopropene (1-MCP; 1 µL L-1 for 12 h at 15 °C [1-MCP]) or without 1-MCP (air [control]) prior to storage and subjected to two postharvest storage scenarios: i) 15 days at 0.5 °C and 85 % relative humidity (RH), followed by 5 days at 5.5 °C, and ii) 20 days at 5.5 °C and 85 % RH. The vertical stripped line shows when all samples were stored at 5.5 °C. Data represents means (n = 90) ± standard error. Least Significant Difference (LSD) for relevant interactions were as follows: LSD_0.05_ [lightness] = 0.92, 0.58 for storage time and temperature, respectively. LSD_0.05_ [_C*, H°_] for storage time = 0.75 and 1.84, respectively.

**Supplementary Figure 2. Abscisic acid (ABA) catabolites (7-dihydroxy-ABA [7-OH-ABA] and dihydrophaseic acid [DPA]) expressed as ng g-1 DW in the proximal (left) and distal (right) sections of ´Krissy´ grapes.** Table grapes were treated with 1-methylcyclopropene (1-MCP; 1 µL L-1 for 12 h at 15 °C [1-MCP]) or without 1-MCP (air [control]) prior to storage and subjected to two postharvest storage scenarios: i) 15 days at 0.5 °C and 85 % relative humidity (RH), followed by 5 days at 5.5 °C, and ii) 20 days at 5.5 °C and 85 % RH. The vertical stripped line shows when all samples were stored at 5.5 °C. Data represents means (n = 90 berries) ± standard error. Least Significant Difference (LSD) bar (P < 0.05) shown when the interaction treatment x temperature x section x storage time was significant. LSD_0.05 [7-OH-ABA]_ section = 2.77.
